# The *Drosophila *Netrin receptor frazzled/DCC functions as an invasive tumor suppressor

**DOI:** 10.1186/1471-213X-11-41

**Published:** 2011-06-14

**Authors:** Adrienne VanZomeren-Dohm, Joseph Sarro, Ellen Flannery, Molly Duman-Scheel

**Affiliations:** 1 Department of Medical and Molecular Genetics, Indiana University School of Medicine, Raclin-Carmichael Hall, 1234 Notre Dame Ave., South Bend, IN 46617, USA; 2 Department of Biological Sciences, Harper Cancer Institute, and Eck Institute for Global Health, University of Notre Dame, Notre Dame, IN 46556, USA

## Abstract

**Background:**

Loss of heterozygosity at 18q, which includes the *Deleted in Colorectal Cancer (DCC) *gene, has been linked to many human cancers. However, it is unclear if loss of *DCC *is the specific underlying cause of these cancers. The *Drosophila *imaginal discs are excellent systems in which to study *DCC *function, as it is possible to model human tumors through the generation of somatic clones of cells bearing multiple genetic lesions. Here, these attributes of the fly system were utilized to investigate the potential tumor suppressing functions of the *Drosophila DCC *homologue *frazzled (fra) *during eye-antennal disc development.

**Results:**

Most *fra *loss of function clones are eliminated during development. However, when mutant clone cells generated in the developing eye were rescued from death, partially differentiated eye cells were found outside of the normal eye field, and in extreme cases distant sites of the body. Characterization of these cells during development indicates that *fra *mutant cells display characteristics of invasive tumor cells, including increased levels of phospho-ERK, phospho-JNK, and Mmp-1, changes in cadherin expression, remodeling of the actin cytoskeleton, and loss of polarity. Mutation of *fra *promotes basement membrane degradation and invasion which are repressed by inhibition of Rho1 signaling. Although inhibition of JNK signaling blocks invasive phenotypes in some metastatic cancer models in flies, blocking JNK signaling inhibits *fra *mutant cell death, thereby enhancing the *fra *mutant phenotype.

**Conclusions:**

The results of this investigation provide the first direct link between point mutations in *fra/DCC *and metastatic phenotypes in an animal model and suggest that Fra functions as an invasive tumor suppressor during *Drosophila *development.

## Background

Loss of heterozygosity (LOH) at chromosome 18q, which includes the *DCC *gene, was identified in a large percentage of colorectal cancers [1]. LOH at 18q is associated with decreased *DCC *expression and has been linked to many other types of cancer, including neuroblastomas, hematologic malignancies, and gastric, endometrial, prostate, ovarian, esophageal, testicular, breast, and glial cancers [2,3]. It was therefore hypothesized that DCC functions as a tumor suppressor. However, although elevated levels of the DCC ligand Netrin (Net) have been linked to oncogenic phenotypes [4,5], point mutations in *DCC *have not been directly associated with tumorigenesis in animal models [2]. For example, loss of *DCC *is not associated with tumor formation in a murine model [6]. However, although the impact of mutating *DCC *in every cell of an organism has been investigated [6], somatic *DCC *mutant clones have not been assessed for cancer phenotypes in animal models. Such an analysis may provide an effective simulation of human tumors and offer insight into the putative tumor suppressing functions of DCC.

*Drosophila melanogaster *is an excellent system in which to study tumor suppressor gene function [7,8]. Many of the hallmarks of human cancer, including self-sufficiency in growth and proliferative signals, insensitivity to anti-proliferative signals, evasion of apoptosis, and invasion/metastasis can be found in *Drosophila *[7]. Mutagenesis screens in *Drosophila *have led to the identification of cancer genes, and studies in flies led to the discovery of important interactions between signaling pathways that function during oncogenesis [7,9,10]. One great advantage of the fly system which is of particular relevance to this investigation is that it is relatively simple to use the FLP/FRT system [11] to create clones of genetically distinct somatic cells that model human tumors. Furthermore, since multiple mutations typically cooperate to generate metastatic tumors, it is important to use animal models such as *Drosophila *in which multiple genetic manipulations can be performed simultaneously when clones are generated.

It was hypothesized that exploitation of the advantages of the *Drosophila *system to study DCC/Fra function would provide new insights into the function of this gene in oncogenesis. Our analysis of *fra *loss of function point mutations during fly development suggest that Fra functions as a tumor suppressor. The results of this investigation directly link loss of function point mutations in *fra/DCC *to metastatic phenotypes in an animal model for cancer.

## Results And Discussion

### Death of *fra *mutant clone cells

**Figure 1:**
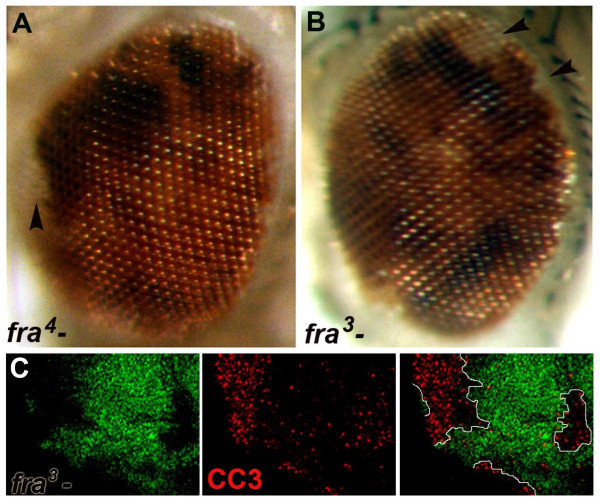
**Death of *fra *mutant clone cells**. Most *fra*^*4 *^(A) and *fra*^*3 *^(B) mutant eye cells generated during development do not persist through adulthood. The few that survive tend to cluster at the periphery of the adult eye (light-pink *w- *cells marked by arrowheads in A and B). Elevated levels of cleaved caspase-3 (CC3, red) are observed in *fra*^*3 *^mutant clones (circled, GFP-negative) generated in the third instar antennal disc (C). Clones were generated with a *hsFLP *driver in A and B, and with *eyFLP *in C and D.

*fra *is expressed in imaginal discs [12], suggesting that Fra might function during imaginal disc development. To test this idea, somatic *fra *loss of function (LOF) clones were generated during eye development. Two different EMS null alleles, *fra*^*3 *^and *fra*^*4*^, both previously shown to lack Fra protein expression [13] were studied in this manner. When either *fra*^*3 *^or *fra*^*4 *^mutant clones are induced during eye disc development, most mutant clones fail to persist through adulthood (Figure 1A,B). In third instar eye discs, *fra*^*3 *^and *fra*^*4 *^mutant clones are typically very small. Staining with anti-cleaved caspase-3, a marker for apoptotic cells, reveals dying *fra *mutant clone cells in the eye-antennal disc (Figure 1C). The majority of *fra *mutant eye clones are eliminated by the end of the third instar.

### Analysis of *fra *loss of function clones in adult flies

**Figure 2:**
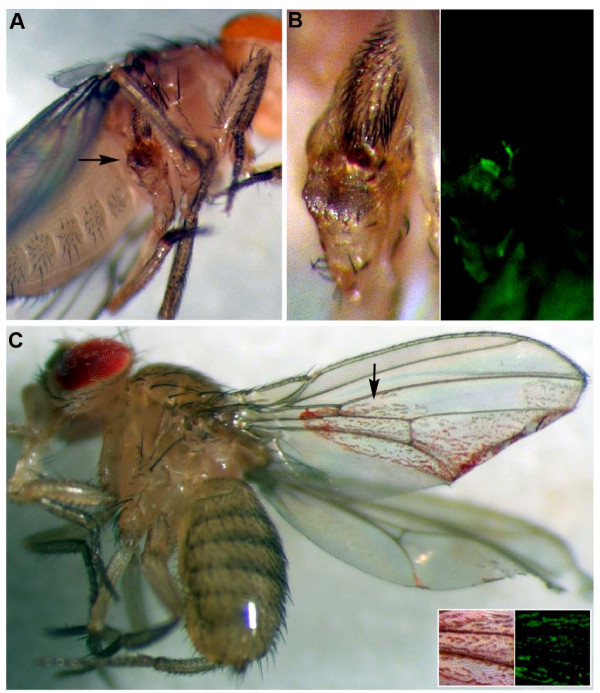
**Tumor-like phenotypes are observed in adult flies when *fra *loss of function clones survive during development**. *fra*^*3 *^mutant tumor-like growths (arrow in A) are detected in a small percentage of animals in which clones are generated during development. A high magnification view of the growth present on an adult leg (arrow in A) is provided in B; lack of GFP expression in this growth marks *fra *mutant cells in the panel at right). Although most animals in which P35-rescued *fra*^*4 *^mutant clones are generated do not survive to adulthood, interesting phenotypes are observed in escaper flies (C). P35-rescued *fra*^*4 *^mutant cells generated in the eye (marked by expression of GFP, red in color due to presence of *w+ *transgenes) are detected in the wing (C, region marked by arrow is magnified at right). Clones were generated with a *hsFLP *driver in A and B and with *eyFLP *in C.

Although most *fra *mutant clones do not persist, clones occasionally survive into the adult stages. In ~1% of adult flies in which *fra*^*3 *^mutant clones are generated (n = 500), mutant clone cells form overgrowths (Figure 2A,B). These observations suggested that it would be useful to develop a strategy that would permit more efficient analysis of *fra *LOF clones. Viability of *fra*^*4 *^mutant cells was increased through expression of the baculovirus caspase inhibitor P35 in mutant clones. In these experiments, the MARCM system [14,15] was used to drive P35 expression in *fra*^*4 *^mutant clones in the developing eye. Generation of P35-rescued *fra*^*4 *^mutant clones typically results in death of the organism (~99% of flies with the appropriate genotype for generating these clones die before eclosion; 2000 F1 progeny were scored from the appropriate P1 cross for generating this genotype). However, interesting phenotypes, which are described in detail below, were observed in 100% of the adult escaper flies that managed to survive to adulthood.

**Figure 3:**
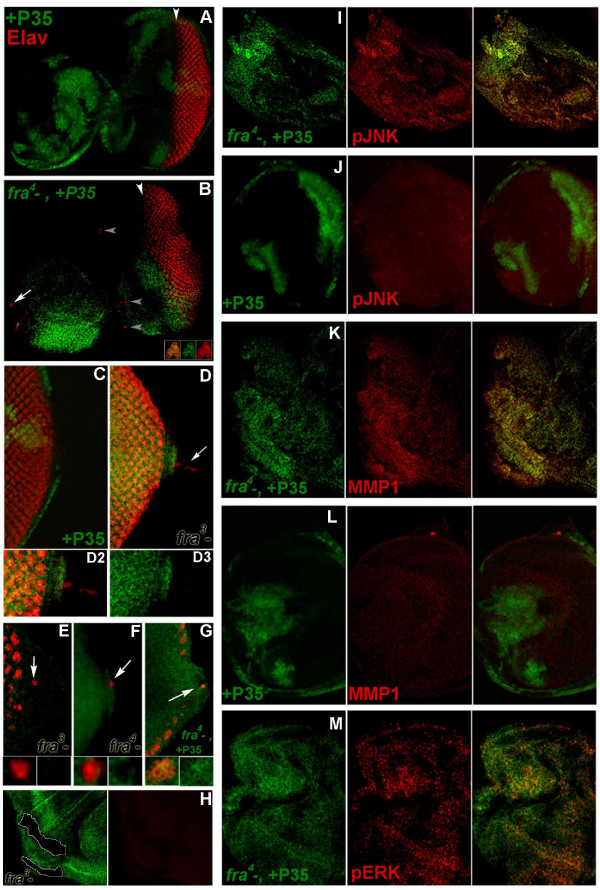
**Characterization of *fra *mutant cells in the developing eye-antennal disc**. Mutant cells are positively marked (green) in A,B,C,G,I,J,K,L,M and negatively marked by lack of GFP in D,E,F,H. During normal eye development, Elav (red in A,B,C,D,E,G,H) is detected in differentiating neurons posterior to (right of) the morphogenetic furrow (white arrowhead, A), but not in the antennal region (left of arrowhead, A). Isolated P35-rescued *fra*^*4 *^mutant cells are found anterior to the furrow (grey arrowheads, B; cells marked by arrow are magnified in inset) and on the optic stalk (arrow, G). Expression of P35 alone (green in A, C) does not produce this phenotype. In the absence of P35-rescue, isolated *fra*^*3 *^(GFP-negative in D,E) or *fra*^*4 *^(GFP-negative in F) cells (arrows) expressing photoreceptor markers (Elav in D,E; Delta in F) invade the optic stalk. GFP-negative *fra*^*3 *^mutant clones (circled) generated in the wing disc do not express Elav (H). P35-rescued *fra*^*4 *^mutant cells (green in I,K,M) express high levels of phospho-JNK (red in I), phospho-ERK (red in M), and Mmp1 (red in K), none of which are altered by P35 alone (J,L). P35-rescued *fra*^*4 *^mutant cells were detected with a P35 antibody (green staining in B,G,I,K) or by GFP expression (M). Control clones expressing P35 alone are marked by GFP (A,C,J,L). *eyFLP *was used to generate clones in B,D,F,G,I,K,M, and *hsFLP *was used in A,C,E,H,J, and L. Third instar eye-antennal discs are oriented anterior left and dorsal up in all panels but H, which shows the posterior ventral portion of the wing disc. The entire eye-antennal disc is shown in A and B, the optic stalk in C,D,E,F,G, and the antennal portion of the disc in I,J,K,L,M.

Perhaps the most striking phenotype observed in flies in which P35-rescued *fra*^*4 *^mutant clones are generated in the eye is the detection of eye cells throughout the bodies of adult flies. One example is shown if Figure 2C, in which GFP-marked *fra *mutant clone cells generated during eye development with the *eyFLP *driver are located on the wing of the adult. Mutant eye cells appear to differentiate, at least in part, as they express the neural differentiation marker Elav in third instar discs (Figure 3B,G) and display red pigmentation in adults (Figure 2C). This intriguing phenotype is not observed in conjunction with P35 ectopic expression alone (not shown). Thus, experiments with two different LOF *fra *alleles suggest that Fra may have tumor-suppressing functions in *Drosophila*, a hypothesis that was explored in more detail during the course of this investigation.

### Analysis of *fra *mutant cells during eye development

The initial results obtained in adult flies suggested that *fra *mutant eye cells might have the capacity to invade surrounding tissues. To assess this possibility, *fra *LOF clones were analyzed during larval development. These analyses were performed in the developing eye-antennal disc, where clones were generated with *eyFLP*. Although the *ey *driver used in this investigation (see methods) was designed to be very tight, occasional FLP expression has been reported in the brain and gonads [16]. We therefore avoided analyses in these tissues and restricted all analyses to the eye-antennal disc so that the point of mutant clone origin would be certain. When *fra*^*3 *^(Figure 3D), *fra*^*4 *^(Figure 3F), or P35-rescued *fra*^*4 *^(Figure 3B,G) LOF clones were generated in the developing eye, a small number of *fra *mutant photoreceptor cell bodies, typically restricted to the eye field (Figure 3A,C), were detected in the optic stalk (Figure 3D,E,F,G) and other inappropriate regions of the eye-antennal disc (Figure 3B). In such cases, *fra *mutant Elav-positive foci were isolated and often located outside of mutant clone boundaries (Figure 3B,D,E,G). Comparable results were obtained when *FLP *was expressed under heat shock control (Figure 3E).

While these results suggested that individual *fra *mutant cells might become invasive and leave the clone, one could alternatively argue that *fra *mutant cells outside of the eye field have undergone a transformation toward an eye cell fate. Several pieces of data indicate that this is not the case. First, when P35-rescued *fra *mutant clones are induced in the antennal disc, there is no evidence of a general conversion of these mutant cells to eye cell fates, as the majority of cells in P35-rescued *fra*^*4 *^antennal clones do not express the photoreceptor marker Elav (Figure 3B). Furthermore, cells of *fra *mutant clones located anterior to the furrow do not appear to undergo precocious differentiation as photoreceptor cells (Figure 3B). Finally, generation of *fra*^*3 *^mutant clones in the wing disc with *hsFLP *does not promote transformation of these cells toward an Elav-positive photoreceptor fate (Figure 3H); this result indicates that the *fra *mutant cells located on the adult wing (Figure 2C) are not simply the result of any rare leaky expression of FLP inducing a *fra *mutant wing clone that is transformed into eye tissue. Together, these data suggest that eye cells located outside of the eye field do not result from transformation of other tissues toward an eye fate.

### Loss of *fra *in the developing eye results in expression of invasive cell markers and altered polarity, adhesion, cytoskeletal organization, and proliferation

**Figure 4:**
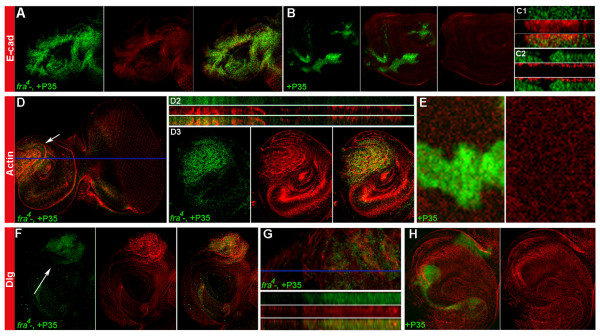
**Somatic mutation of *fra/DCC *produces changes in adhesion, the cytoskeleton, and polarity**. Mutant clones/cells are marked in green in all panels. E-cad (red) is not properly localized to the membrane in P35-rescued *fra*^*4 *^mutant clone cells (antenna shown in A; orthogonal section of antennal clone shown in C1). E-cad is not altered by expression of P35 alone (antenna shown in B, orthogonal section through antennal clone shown in C2). Changes in the Actin cytoskeleton (red, D) are observed in a P35-rescued *fra*^*4 *^mutant clone in the antennal disc (arrow). The region marked by the arrow in D is magnified in D3, and an apical up orthogonal section through the disc is shown in D2 (blue line in D marks location of section). Furthermore, P35-rescued *fra*^*4 *^mutant cells display delocalization of the basolateral marker Dlg (red in antennal clones in F,G; apical up orthogonal section of region marked by blue line in G is shown in lower portion of panel). E-cad (red in B,C2), Actin (red in E), and Dlg (red in H) expression are normal in control clones expressing ectopic P35 alone, which are marked by GFP. P35-rescued *fra*^*4 *^mutant cells were marked by GFP (A,D) or detected with a P35 antibody (green in F,G). Third instar discs are oriented anterior left and dorsal up in all panels. All *fra *mutant clones were generated with the *eyFLP *driver.

*fra *mutant eye-antennal disc clones were next assessed for molecular features typically associated with invasive cell types. Although use of P35-rescue was often utilized to permit analysis of larger clones generated with the *eyFLP *driver, the use of P35 was experimentally controlled for throughout the investigation. Phospho-JNK (Figure 3I), Mmp1 (Figure 3K), and phospho-ERK (Figure 3M), all of which are upregulated in other *Drosophila *invasive tumor models [17], are elevated in P35-rescued *fra*^*4 *^mutant clones. Mutant cells were examined for potential changes in adhesion, cytoskeletal organization, and polarity, additional features of metastatic cell types. Within P35-rescued *fra*^*4 *^mutant clones, E-cad expression is slightly increased, yet delocalized from the membrane (Figure 4A,C1). However, little E-cad expression is detected in individual *fra *mutant cells that are found outside of clone boundaries (not shown). Changes in cytoskeletal organization are observed in *fra *mutant cells, as illustrated by alterations in the actin cytoskeleton detected in a P35-rescued *fra*^*4 *^clone (Figure 4D). None of these phenotypes were found in clones expressing P35 alone (Figure 3J,L, Figure 4B,C2,E).

**Figure 5:**
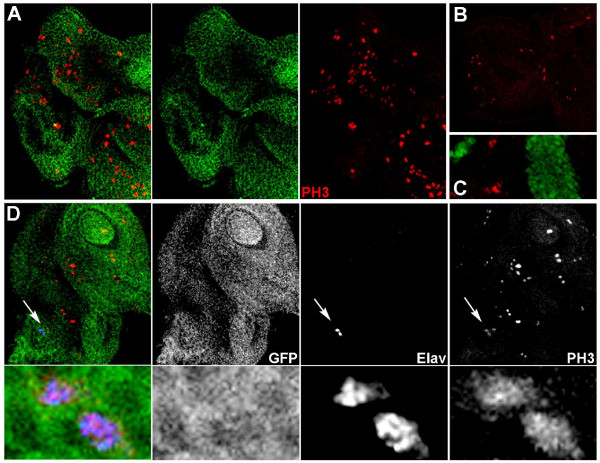
**Somatic mutation of *fra/DCC *results in increased cell division**. P35-rescued *fra*^*4 *^mutant clone cells are marked by GFP throughout this figure. Mitotic cells are marked by red anti-phosphorylated Histone H3 staining. An increased number of mitotic cells is observed in P35-rescued *fra*^*4 *^mutant clone cells (antenna and anterior portion of the eye shown in A); fewer mitotic cells are observed in a comparable region in a wild-type disc (B). Such excess cell division does not result from overexpression of P35 alone (green, C). In D, two Elav-positive cells located in the antenna (blue, marked by arrow) display elevated phosphorylated Histone H3 levels (overlay shown at left in D, and three single channel pictures are shown at right; high magnification views of the two cells are shown in the lower panels). Clones were generated with the *eyFLP *driver in A and D and with the *hsFLP *driver in C. Discs are oriented anterior-left and dorsal upwards, except for the disc in D, in which the dorsal side is oriented approximately 35° right of top center.

Polarity changes were assessed through analysis of Discs-large (Dlg), a basolateral marker and neoplastic tumor suppressor [18]. P35-rescued *fra*^*4 *^mutant cells display delocalization of Dlg (Figure 4F,G), suggesting that loss of *fra *results in disrupted apical-basal cell polarity. Disruption of apical-basal polarity is often accompanied by changes in cell proliferation [18]. Increased expression of the mitotic marker phosphorylated Histone H3 is also detected in P35-rescued *fra*^*4 *^mutant cells (Figure 5A; compare to Figure 5B). Expression of this mitotic marker was even observed in ~10% of Elav-positive *fra *mutant cells located in the antennal disc, far outside of the normal photoreceptor field (Figure 5D, n = 20). Again, neither changes in polarity nor proliferation were found in clones expressing P35 alone (Figure 4H, Figure 5C). In summary, characterization of *fra *mutant cells during eye-antennal disc development indicates that they display several features that are characteristic of metastatic tumor cells.

### Loss of *fra *promotes basement membrane degradation and invasion

**Figure 6:**
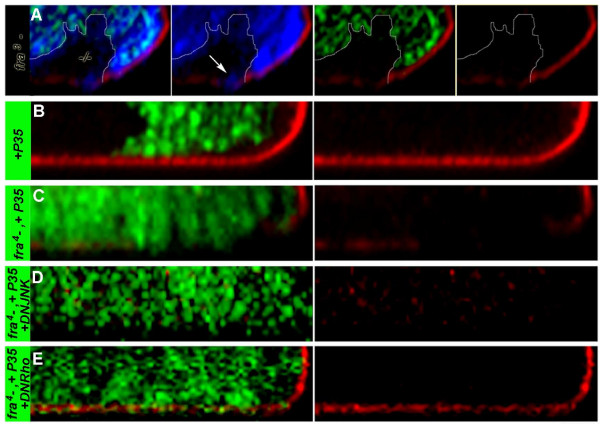
**Loss of *fra *results in Rho1-mediated basement membrane degradation and invasion**. GFP expression marks mutant cells in B,C,D,E, and mutant cells are marked by lack of GFP expression in A. The basement membrane (marked by Perlecan staining, red in all panels) is degraded in regions where *fra*^*3 *^mutant clones (circled GFP-negative clone, A; nuclear stain is shown in blue) or P35-rescued *fra*^*4 *^mutant clones (green, C) are generated (compare to green control clones expressing P35 alone which do not disrupt the basement membrane in B). *fra*^*3 *^(A) and P35-rescued *fra*^*4 *^mutant cells (C) invade the basement membrane (compare to control in B). Neither invasion nor degradation of the basement membrane is blocked by coexpression of dominant negative-JNK in *fra*^*4 *^mutant cells (green, D). Coexpression of DN-Rho1 in *fra*^*4 *^mutant clone cells (green, E) partially rescues basement membrane degradation and cell invasion. Clones were generated with the *eyFLP *driver in all panels except B, in which clones were generated with *hsFLP*. Orthogonal sections through the posterior dorsal edge of eye discs are oriented apical up in all panels.

Several features of *fra *mutant cells, including changes in adhesion, polarity, cytoskeletal organization, and activation of JNK and Mmp-1 (Figures 3 and 4), are consistent with basement membrane invasive tumor models in *Drosophila *[7,19,20]. We therefore tested the impacts of *fra *LOF on basement membrane degradation and invasion in the developing eye disc. The basement membrane was degraded adjacent to both *fra*^*3 *^(Figure 6A) and P35-rescued *fra*^*4 *^mutant clones (Figure 6C). Unlike control cells (Figure 6B), both *fra*^*3 *^(Figure 6A) and P35-rescued *fra*^*4 *^mutant cells (Figure 6C) were found to lose epithelial polarity and invade the basement membrane.

Live imaging experiments captured the invasive properties of *fra *mutant cells. GFP-positive P35-rescued *fra*^*4 *^mutant cells detected just outside of the eye-antennal disc were imaged in live tissue preparations. In the resulting videos (Additional File 1 is a still frame reference for the movies in Additional Files 2 and 3), these GFP-positive cells are motile. At higher magnification (Additional File 3), projections that extend and retract as the cell migrates are observed. Such motility was never observed in response to expression of P35 alone. These data indicate that *fra *mutant cells exhibit invasive properties.

### Inhibition of Rho but not JNK signaling suppresses basement membrane degradation and invasion of *fra *mutant cells

**Figure 7:**
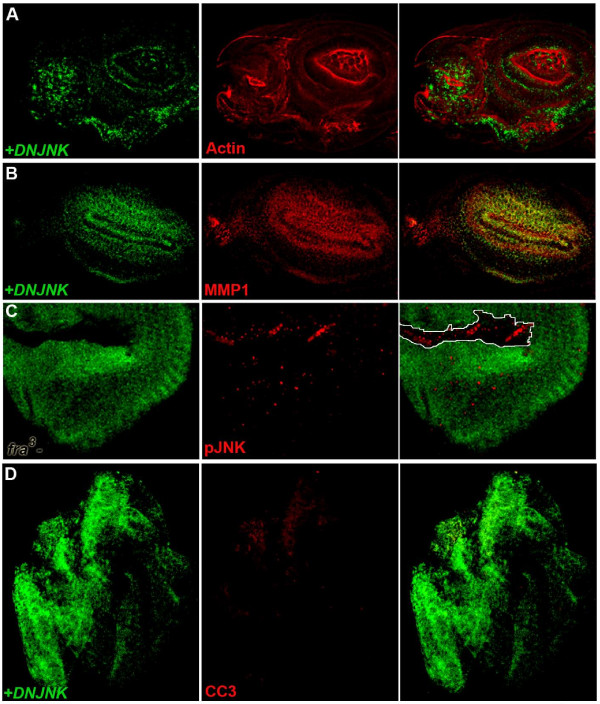
**Inhibition of JNK signaling enhances the *fra *mutant phenotype**. Dominant-negative JNK was expressed in P35-rescued *fra*^*4 *^mutant clones (green in A,B,D,E). Such clones have disrupted Actin staining (red in A, antenna shown) and ectopically express MMP-1 (red in B, antenna shown), but have little anti-cleaved caspase-3 staining (red, D). Thus, although blocking JNK signaling does not prevent *fra *mutant cell invasion, inhibition of JNK, which is activated in non-rescued *fra*^*3 *^mutant clones (circled, GFP-negative in C), blocks cell death. Such inhibition of JNK can result in highly dysmorphic discs (entire eye-antennal disc shown in D). Discs are oriented anterior left and dorsal upwards. Clones were induced with the *eyFLP *driver.

Inhibition of JNK signaling through expression of dominant negative-JNK is known to prevent basement membrane degradation and cell invasion in other fly metastatic tumor models [7,19,20]. However, expression of DN-JNK in P35-rescued *fra*^*4 *^mutant clones did not prevent the actin cytoskeleton (Figure 7A) or E-cad expression/localization (not shown) changes, Mmp-1 expression (Figure 7B), basement membrane degradation, or cell invasion (Figure 6D) associated with loss of *fra*. Furthermore, no adult animals in which such clones had been generated were recovered in this investigation.

Rho1 activation has also been associated with invasive cell behavior in flies [16,21]. Expression of DN-Rho1 in P35-rescued *fra*^*4 *^mutant clones repressed basement membrane degradation and partially suppressed *fra *mutant cell invasion (Figure 6E). These results suggest that loss of *fra *promotes Rho1-mediated basement membrane degradation and cell invasion. Although repression of basement membrane degradation and invasion of *fra *mutant cells in which Rho signaling had been blocked was substantial, it was not complete (Figure 6E), and expression of Mmp-1 was not entirely blocked (not shown). Likewise, co-expression of DN-Rho did not block organismal lethality, as no adult animals in which clones had been generated were recovered.

### Inhibition of Jnk Signaling enhances the *fra *mutant phenotype

Although blocking JNK signaling is known to prevent basement membrane degradation and cell invasion in fly metastatic tumor models [7,19,20], as discussed above, it did not prevent basement membrane degradation or cell invasion associated with loss of *fra *(Figure 6D). In fact, analysis of the impact of inhibiting JNK signaling in *fra *mutant clones suggested that blocking JNK signaling actually enhances the *fra *LOF phenotype. This is illustrated by the highly dysmorphic overgrowths that can result when JNK signaling is blocked in *fra *LOF clones (Figure 7D).

Bossuyt et al. [22] recently demonstrated that tumorous *eyeful *cells activate a JNK-mediated stress response pathway that results in cell suicide. It therefore seemed possible that clonal loss of *fra*, which results in cell death (Figure 1), might occur in response to JNK activation. Indeed, *fra*^*3 *^mutant clones that have not been rescued from lethality exhibit increased levels of phosphorylated JNK (Figure 7C). Little cleaved caspase-3 expression is detected when JNK signaling is blocked in P35-rescued *fra *mutant clones (Figure 7D). These results suggest that cell death associated with non-rescued *fra *LOF clones (Figure 1A-C) is induced by JNK.

## Conclusions

### Direct connection between loss of function point mutations in *fra/DCC *and cancer-like phenotypes

Although mutations in human chromosome 18q, which includes *DCC *and a number of other genes, have been linked to many human cancers [2,3,8], it is unclear if loss of *DCC *is the specific underlying cause of these cancers, as point mutations in *DCC *had not been directly associated with cancer phenotypes in animal models [2]. The results of this investigation provide the first direct link between LOF point mutations in *fra/DCC *and cancer-like phenotypes in an animal model. It is interesting to consider the results of this investigation in relation to the *DCC *knockout mouse [6], which does not have a higher incidence of tumors. In addition to DCC, several other proteins function as Net receptors in vertebrates [2,3], suggesting that redundancy could explain the lack of cancer phenotypes in the *DCC *knockout mouse. However, recent studies have highlighted the importance of studying cancer genes in a clonal context [16]. It is therefore possible that using an experimental design more comparable to the one employed here, one in which clones of mutant *DCC *cells are generated and rescued from death, could reveal cancer phenotypes in mice.

Although many of the results of this investigation are consistent with other metastasis models in flies [19,20,23], it should be noted that comparable adult phenotypes in which eye cells are detected outside of the eye field of the adult are not typically reported. One exception is the *eyeful *study described by Ferres-Marco *et al*. [24]. A potential explanation for the lack of similar phenotypes is that such adult flies, at least in the case reported here, are rarely viable. It should also be noted that the *eyeful *phenotype, like the P35-rescued *fra *mutant phenotype, was the result of several simultaneous genetic manipulations. These studies underline the importance of using genetically tractable animal models wherein such complicated genetic manipulations are possible.

It should be noted that although P35 has been associated with conferring an "undead" cell fate in *Drosophila*, (see [25,26] ), we do not believe that use of P35 was a confounding factor in this investigation. First, no non-cell autonomous impacts were observed. Also, data demonstrating the invasive qualities of non-P35-rescued *fra*[3] and *fra*[4] mutant clones were included. Non P35-rescued mutant cells invade the optic stalk (Figure 3D,F). Basement membrane degradation and invasion is not dependent on P35-rescue of clones (Figure 6A). Finally, an example in which a non-rescued clone has resulted in a tumor (Figure 2A,B) is included. Rescue with P35 was a useful tool, as it increased the number and size of mutant clones that could be analyzed.

### Mutations in *fra/DCC *promote Rho1-mediated invasion

Developmental characterization of *fra *mutant cells indicates that they express a number of tumor cell markers (Figure 3I,K,M) and exhibit changes in expression of E-cad (Figure 4A,C1), reorganization of the actin cytoskeleton (Figure 4D), and loss of apical-basal polarity (Figure 4F,G), characteristics which are typical of invasive tumor cells. *fra *mutant cells also overproliferate (Figure 5A,D). Furthermore, loss of *fra *results in basement membrane degradation and invasion (Figure 6A,C). A proportion of these invasive cells can divide, even if they retain expression of the neural differentiation marker Elav (Figure 5D). These data, in conjunction with live imaging assays (Additional Files 1, 2, and 3), support the notion that loss of *fra *results in a metastatic phenotype.

Although it was hypothesized that JNK, a mediator of metastasis [27] that is upregulated in *fra *mutant cells (Figures 3I, 7C), might drive their invasion, inhibition of JNK signaling did not suppress basement membrane degradation or invasion of *fra *mutant cells (Figure 6D). Instead, inhibition of JNK signaling in *fra *mutant cells appears to block JNK-mediated cell death (Figure 7C,D), resulting in enhanced overgrowth of *fra *mutant cells (Figure 7D) which retain their invasive qualities (Figure 6D). These results are somewhat surprising, as JNK-induced Mmp1-dependent degradation of the basement membrane was identified as a critical early event during the invasion process of metastasizing cells [27]. However, several groups have recently demonstrated that JNK function is context dependent, and that JNK activity does not always function to promote cell invasion [22,28]. Additionally, JNK-mediated apoptosis has been associated with a number of other tumor suppressor mutations in *Drosophila *(see discussion in [28]), and it is therefore not unexpected that it would play a key role in the removal of *fra *mutant clone cells. In future experiments, it will be useful to determine if inhibition of JNK signaling in the absence of P35 rescue is sufficient to prevent cell death in *fra *mutant clone cells.

Rho1 has also been associated with invasive cell behavior in the wing and eye imaginal discs [16,21]. Rho proteins are important regulators of cell shape, motility, and cytoskeletal arrangements that drive epithelial-mesenchymal transitions [29]. Inhibition of *Rho1 *signaling in *fra *mutant cells represses basement membrane degradation and invasion (Figure 6E). These results suggest that Rho1 can induce *fra *mutant cell invasion independently of JNK, at least in an eye developmental context. Repression of basement membrane degradation and invasion by DN-Rho1 was substantial, though not complete. This could result from incomplete deactivation of Rho1 signaling, but might also signify that basement membrane degradation and invasion could be mediated by additional factors. In support of this idea, Mmp-1 expression is not entirely suppressed when Rho1 signaling is blocked in *fra *mutant clones. We are currently pursuing global analysis of gene expression in *fra *mutant cells, which may elucidate additional molecules that are involved. It will be interesting to determine if other signaling pathways, such as the Hippo pathway which has attracted a great deal of attention in recent *Drosophila *imaginal disc studies [22,28,30], is important in the *fra *mutant clonal cell context.

### Implications for the DCC dependence receptor model

DCC is proposed to function as a dependence receptor that induces cell death in the absence of Net ligand. In the presence of Net, which is expressed in many types of tumors, cells are proposed to escape death through downregulation of DCC [2,5,31]. In support of the DCC dependence receptor model, Mazelin *et al*. [32] demonstrated that ectopic expression of Net-1 in the mouse gastrointestinal tract results in spontaneous formation of hyperplastic and neoplastic lesions. They also showed that overexpression of Net-1 in an *APC *mutant background promotes intestinal tumor development by blocking DCC-induced apoptosis [32]. More recently, Fitamant *et al*. [5] showed that Net-1 promotes cell survival in breast cancer cells. These studies support the theory that DCC functions as a dependence receptor with respect to its ability to induce apoptosis. Based on the dependence receptor model, one might have expected that loss of function mutations in *fra *would result in increased cell viability. However, in the *Drosophila *eye/antennal disc, most loss of function *fra *clones do not persist (Figure 1). One possible interpretation of these data in light of the recent Net literature is that Net ligand, which is expressed throughout the developing eye-antennal disc [12], provides a survival cue for developing cells in this tissue; *fra *mutant cells would lack the ability to receive this survival cue and ultimately die in response to JNK activation (Figure 7C).

It is also interesting to consider the dependence receptor model in terms of metastasis. If the dependence receptor model is applicable to metastasis, DCC would be expected to function as a suppressor of metastasis, and as supported by this investigation, mutation of *DCC/fra *would be expected to promote metastasis. According to the dependence receptor model, binding of Net ligand to DCC would inhibit its ability to suppress metastasis, and an abundance of Net ligand would therefore promote metastasis. Several recent studies have demonstrated that Net-1 does promote metastasis. Rodrigues *et al*. [33] described metastatic phenotypes associated with Net-1 activation in human colorectal cancer cells. Furthermore, Fitamant *et al*. [5] found that in comparison to non-metastatic breast tumors, Net-1 levels are elevated in a large proportion of metastatic breast cancers. Their study showed that reduction of Net-1 signaling inhibits metastasis in a mouse model of lung colonization of a mammary cancer cell line, as well as in a model of lung metastasis in xenografted human breast tumors. Net-1 therefore induces metastasis of several types of cancer cells. Taken together, the results presented here in conjunction with these other studies suggest that DCC functions as a suppressor of metastasis, a function that is inhibited by an abundance of Net ligand which can suppress DCC and promote invasion. This model may also apply to regulation of the invasive growth of axons during neural development.

## Methods

### Drosophila Genetics

For eye-specific clone induction, the *eyFLP *construct described in [34], which consists of a 258 bp eye-specific enhancer fragment from the *ey *gene, was used. Generation of hsFLP-induced clones was performed as described previously [9]. In short, animals were heat-shocked for 10-15 minutes at roughly 48 hours after egg-laying, and clones were assessed in the late third instar. Genotypes of the flies used in this investigation were as follows:

*w eyFLP *or *w hsp70-FLP*; *P{FRT(w[24]}G13 P{Ubi-GFP.nls}2R1 P{UbiGFP.nls}2R2/P{w[+mW.hs]=FRT(w[hs])}G13 fra* and *w eyFLP *or *w hsp70-FLP*; *P{FRT(w[24])}G13 P{Ubi-GFP.nls}2R1 P{UbiGFP.nls}2R2/P{w[+mW.hs]=FRT(w[hs])}G13 fra[3]*. P35-rescued *fra[4]*mutant clones were generated via the MARCM system [13,14] in flies of the following genotypes: *eyFLP; P{FRT(w[24])}G13 P{piM}45F P{tubP-GAL80}LL2/P{w[+mW.hs]=FRT(w[hs])}G13 fra[4]*; *P{tubP-GAL4}LL7/UAS-p35.H.BH3 *(clones were detected with an anti-P35 antibody) or *eyFLP; P{FRT(w[24])}G13 P{piM}45F P{tubP-GAL80}LL2/P{w[+mW.hs]=FRT(w[hs])}G13 fra[4]*; *P{tubP-GAL4}LL7/UAS-p35.H.BH3 UAS-EGFP-34 *(clones were positively marked by GFP expression). Rescue by DN-JNK or DN-Rho1 was assessed in the following genotypes: *eyFLP/UAS-DN-JNK; P{FRT(w[24])}G13 P{piM}45F P{tubPGAL80}LL2/P{w[+mW.hs]=FRT(w[hs])}G13 fra[4]*; *P{tubP-GAL4}LL7/UAS-p35.H.BH3 UAS-EGFP-34 *and *eyFLP/UAS-DN-Rho1; P{FRT(w[24])}G13 P{piM}45F P{tubPGAL80}LL2/P{w[+mW.hs]=FRT(w[hs])}G13 fra[4]*; *P{tubP-GAL4}LL7/UAS-p35.H.BH3 UAS-EGFP-34*. GFP-positive control clones in which P35 expression was driven ectopically were produced in flies of the following genotype: *hsp70-FLP; GAL4-Act5C(FRT.CD2).P}S UAS-GFP/UAS-P35*. Additional information about all of these fly strains is available at Flybase (http://flybase.bio.indiana.edu).

### Immunohistochemistry

Immunohistochemistry was performed generally as described by Patel [35]. The following antibodies were used in this investigation: anti-GFP (Sigma-Aldrich, St. Louis, MO), anti-Phospho-p44/42 Map kinase (Cell Signaling Technology, Danvers, MI), anti-DCad2, anti-Dlg 4F3, anti-Elav, anti-Delta, anti-Mmp1 antibodies 14A3D2, 5H7B11, 3B8D12, and 3A6B4 (Developmental Studies Hybridoma Bank, Univ. of Iowa), anti-Active JNK (Promega, Madison, WI), anti-P35 (Novus Biologicals, Littleton, CO), anti-Phospho-Histone H3 [pSer10] (Sigma-Aldrich, St. Louis, MO), anti-cleaved caspase-3 (Cell Signaling Techology, Danvers, MA), and anti-Perlecan (provided by S. Baumgartner). Texas Red-X Phalloidin was obtained from Molecular Probes (Eugene, OR). Nuclei were labeled with To-Pro-3 (Invitrogen, Carlsbad, CA). Secondary antibodies were obtained from Jackson ImmunoResearch (West Grove, PA). Imaging was performed on a Zeiss LSM 710 laser scanning microscope. At least 20 discs per genotype were examined. Image processing was completed with Zen 2008/2009 Light and Adobe Photoshop software.

### Live imaging

Live imaging was performed with a Zeiss LSM 710 laser scanning microscope. Third instar larvae were dissected and placed in a drop of saline solution on a microscope slide under a number 1 glass cover slip. Motile GFP-expressing clones were checked for auto-fluorescence, which would rule out that they were hemocytes. In Additional File 2, cells were imaged every 5.0 seconds with a frame average of 1 for ~2.5 minutes. In Additional File 3, the cell was imaged every 1.0 second with a frame average of 1 for ~1 minute. The resulting movies were processed using Zeiss LSM software.

## Authors' contributions

AVD performed genetic crosses, immunohistochemistry and microscopy, analyzed data, and helped draft the manuscript. JS performed live imaging assays and assisted with genetic crosses, data analysis, and drafting the manuscript. EF assisted with experimental design, immunohistochemistry, microscopy, data analysis, and drafting of the manuscript. MDS conceived of the study, participated in its design and coordination, performed experiments, evaluated data, and prepared the manuscript. The authors have read and approved the final manuscript.

## Supplementary Material

Additional file 1 **Still image accompanying live imaging movies of invasive *fra *mutant cells**. A stationary GFP-positive P35-rescued *fra*^*4 *^mutant clone in the ventral portion of the eye disc is marked by an * in the still image. Migratory mutant cells observed in the accompanying live imaging movies are marked by arrows. Click here for file

Additional file 2 **Live imaging of invasive *fra *mutant cells, 55×**. GFP-positive P35-rescued *fra*^*4 *^mutant cells exiting the eye-antennal disc were imaged in live tissue preparations. In this video, two migratory cells (marked by arrows in Figure S1) have exited the ventral portion of the eye disc. Click here for file

Additional file 3 **Live imaging of invasive *fra *mutant cell, 200×**. The migratory P35-rescued *fra*^*4 *^mutant cell marked by the large arrow in the lower portion of Figure S1 is captured at higher magnification. Here, projections extending and retracting as the cell moves can be observed. Click here for file
