# Supplementary figures and images for: The Drosophila Netrin receptor frazzled/DCC functions as an invasive tumor suppressor
Source: BMC Dev Biol. 2011 Jun 14;11:41. doi: 10.1186/1471-213X-11-41 (PMC3144007; doi:10.1186/1471-213X-11-41)

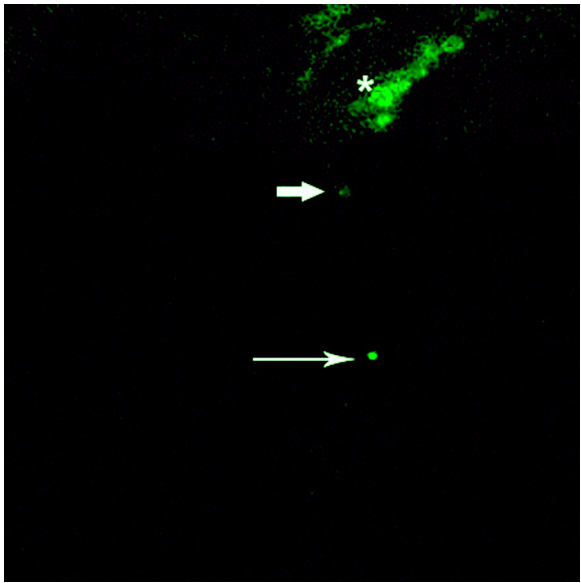

Supplement: Additional file 1 — Still image accompanying live imaging movies of invasive fra mutant cells. A stationary GFP-positive P35-rescued fra4 mutant clone in the ventral portion of the eye disc is marked by an * in the still image. Migratory mutant cells observed in the accompanying live imaging movies are marked by arrows. [file 1471-213X-11-41-S1.PDF]
